# Monitoring the efficacy of the cleaning and disinfection process for flexible endoscopes by quantification of multiple biological markers

**DOI:** 10.1186/2047-2994-4-S1-P56

**Published:** 2015-06-16

**Authors:** M Bommarito, J Stahl, D Morse, H Reuter

**Affiliations:** 1 Infection Prevention Division, 3M, St Paul, MN, USA; 2 Infection Prevention Division, 3M, Neuss, Germany

## Introduction

The contamination found in flexible gastrointestinal endoscopes was characterized at each step of reprocessing, by measuring: Adenosine Tri-Phosphate (ATP), colony forming units (CFUs), protein, and blood. Bacterial species isolated from culture positive scopes were identified.

## Objectives

To understand the effectiveness of the standard cleaning and disinfection process for these complicated devices, and to identify means by which monitoring tools can help define improvements to this process.

## Methods

The concentrations of four markers in 27 colonoscopes and 28 gastroscopes from two different manufactures, reprocessed in the endoscopy suite of a US hospital were measured after bedside flushing, after manual cleaning, and after high level disinfection (HLD). ATP levels in Log(RLUs) (Relative Light Units) were determined using a luminometer. Protein concentrations in μg/mL were determined using a bicinchoninic acid (BCA) assay. Total aerobic Log(CFUs) were determined by culture. Blood concentrations were determined with a colorimetric dipstick. Culture positive scopes were verified by further growth and identification of species using a bioMerioux Vitek 2 Compact system.

## Results

For each reprocessing step, gastroscopes showed significantly higher ATP levels compared to colonoscopes (p<0.0001). The Log reductions in ATP contamination were found to be significant for each reprocessing step, whereas for CFUs the only significant reduction is after HLD. 11% of the gastroscopes were culture positive after HLD. Protein levels were highly correlated with ATP levels. 17% of the gastroscopes had measurable blood pre-manual cleaning and one device was also blood positive after HLD. Species from culture positive scopes identified before manual cleaning were of human origin but after manual cleaning many waterborne organisms were also found.

## Conclusion

Levels of ATP, CFUs, protein and blood found in patient-used endoscopes showed different reductions by type of endoscope and reprocessing step. To define more effective reprocessing strategies and protocols for better infection prevention, understanding contamination levels as well as its composition and how it changes through each reprocessing step, requires monitoring multiple parameters.

## Disclosure of interest

M. Bommarito Employee of: 3M, J. Stahl Employee of: 3M, D. Morse Employee of: 3M, H. Reuter Employee of: 3M.

